# CD4^+^Foxp3^+^ regulatory T cell differentiation mediated by endometrial stromal cell-derived TECK promotes the growth and invasion of endometriotic lesions

**DOI:** 10.1038/cddis.2014.414

**Published:** 2014-10-02

**Authors:** M-Q Li, Y Wang, K-K Chang, Y-H Meng, L-B Liu, J Mei, Y Wang, X-Q Wang, L-P Jin, D-J Li

**Affiliations:** 1 Laboratory for Reproductive Immunology, Hospital and Institute of Obstetrics and Gynecology, Fudan University Shanghai Medical College, Shanghai, China; 2 Shanghai Key Laboratory of Female Reproductive Endocrine Related Diseases, Shanghai, China; 3 Department of Assisted Reproduction, Shanghai Ninth People's Hospital Affiliated Shanghai JiaoTong University School of Medicine, Shanghai, China; 4 Department of Obstetrics and Gynecology, The Fourth Hospital of Soochow University, WuXi, China

## Abstract

Endometriosis is associated with an abnormal immune response to endometrial cells, which can facilitate the implantation and proliferation of ectopic endometrial tissue. The proportion of CD4^+^Foxp3^+^ regulatory T cells (Tregs) is significantly increased in the peritoneal fluid of women with endometriosis. The thymus-expressed chemokine TECK/CCL25 directly promotes the invasiveness of endometrial stromal cells (ESCs). The aim of this study was to investigate the effects of ESC-derived TECK on the crosstalk between Tregs and ESCs in the progress of endometriosis. We determined that the percentage of Tregs and the concentration of TECK increased in the peritoneal fluid with the progression of endometriosis. The supernatant from co-cultured human ESCs and macrophages not only induced Treg differentiation and increased Treg expression of interleukin-10 (IL-10), transforming growth factor-*β* (TGF-*β*) and CD73 by activating the AKT/STAT3 signaling pathway but also repressed Treg apoptosis by downregulating Fas and FasL expression and enhanced the Treg-mediated suppression of CD4^+^CD25^−^ T cells. In addition, *in vitro* and *in vivo* trials confirmed that these effects could be inhibited by anti-TECK neutralizing Abs. The secretion of IL-10 and TGF-*β* by Tregs increased MMP2 expression and decreased TIMP1 expression and further stimulated the proliferation and invasion of ESCs and the growth of ectopic lesions. These results indicate that TECK derived from ESCs and macrophages upregulates the number and function of Tregs in the ectopic milieu, which contributes to endometriotic immunotolerance and high levels of ESC proliferation and invasion, thereby facilitating the progression of endometriosis.

Endometriosis is one of the most common gynecological diseases in women with a prevalence rate of ~10%. It is characterized by the presence of endometrial glands and stroma at extrauterine sites and manifests with pelvic pain and infertility.^1^ Despite decades of intensive investigation, little is known about the pathogenesis of endometriosis. The most widely accepted etiology is Sampson's theory of retrograde menstruation where shed endometrial tissue is refluxed through the fallopian tubes and attaches and proliferates within the pelvis.^2^ However, it is not fully understood why, even though the majority of women have retrograde menstruation, only about one in ten women develop endometriosis. This suggests that other factors may mediate the formation of endometriotic lesions.

Several recent studies have focused on the importance of immunologic imbalances in women with endometriosis. In fact, a permissive peritoneal environment may be associated with a dysregulated immune response to endometrial cells. Instead of effectively removing endometrial fragments at pelvic cavity, this environment can facilitate the implantation, neo-angiogenesis and proliferation of ectopic endometrial tissue.^3,4^ These conditions may include elevated levels of activated peritoneal macrophages, reduced natural killer cell activity and an abnormal T lymphocyte response. Recently, several groups have reported the presence of Tregs in eutopic and ectopic endometrial tissue from patients with endometriosis.^5,6^ In addition, the number of Tregs is significantly increased in peritoneal fluid of women with endometriosis.^7,8^ However, the mechanism behind the increase in the number of Tregs in the peritoneal fluid of women with endometriosis and the role of Tregs in the progression of endometriosis are unknown.

Chemokines produced in the endometriotic milieu may contribute to a feed-forward cascade of events, which promotes the recruitment of leukocytes to the peritoneal cavity and regulates the proliferation and invasion of endometrial stromal cells (ESCs) in patients with endometriosis. These chemokines include Regulated on Activation, Normal T Cell Expressed and Secreted (RANTES), monocyte chemotactic protein and interleukin-8 (IL-8).^9,10^ The thymus-expressed chemokine (TECK/CCL25), which was initially reported to be produced by thymic cells, is highly expressed in endothelial cells and a subset of cells in the small intestine.^11^ CC chemokine receptor 9 (CCR9), previously designated GPR-9-6, as a specific receptor for TECK, is expressed mainly in immature T cells such as double-positive T cells and gut-associated T cells.^12^ The TECK–CCR9 interaction has an important role in regulating T-cell development and tissue-specific homing.^12^ In addition, CCR9-mediated signaling is involved in anti-apoptotic signaling to the T cells.^13^ Interestingly, our previous study confirmed that TECK from a variety of cells in the endometriotic milieu (for example, ESCs, peritoneal mesothelial cells and macrophages) promotes ESC invasion in endometriosis by increasing the expression of metalloproteinase 2/9 (MMP2/9).^14^

Therefore, the aim of this study was to investigate whether TECK in the endometriotic milieu regulates the Treg differentiation, apoptosis and function and to explore further the effect of these educated Tregs on the growth and invasion of ESCs in endometriosis.

## Results

### The ratio of CD4^+^Foxp3^+^ Tregs and TECK concentration in peritoneal fluid is positively correlated with the progression of endometriosis

**Figure 1:**
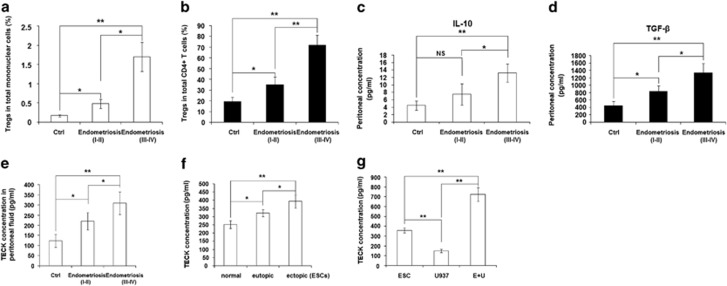
The ratio of CD4^+^Foxp3^+^ Tregs and TECK concentration in peritoneal fluid is positively correlated with the progression of endometriosis. Number of CD4^+^Foxp3^+^ Tregs (**a**, **b**) and the concentration of IL-10 (**c**), TGF-*β* (**d**) and TECK (**e**) in peritoneal fluid from healthy controls (Ctrl) and patients with early stage endometriosis (r-AFS stage I and II) and advanced stage endometriosis (stage III and IV), as determined by flow cytometry and ELISA. (**f**) The level of TECK secreted by primary ESCs isolated from healthy endometrium, eutopic endometrium and ectopic lesions from patients with endometriosis was analyzed by ELISA. (**g**) The level of TECK in the supernatant of ESCs from ectopic lesions from patients with endometriosis, from U937 cells and from co-cultured ESCs (from ectopic lesions) and U937 cells. The cells were cultured for 48 h and TECK secretion was assessed by ESLIA. E+U: co-culture d ESCs and U937 cells. Data are expressed as the mean±S.D. *P*<0.05 was considered to be statistically significant. Values with double asterisks (**P<0.01) are significantly different from those with a signal asterisk (*P<0.05). ns: no statistical difference

Quantitative analysis showed that the percentage of CD4^+^Foxp3^+^ Tregs was significantly increased not only in the total mononuclear cells but also in CD4^+^ T cells of peritoneal fluid from women with endometriosis (stage I–II and stage III–IV) compared with healthy fertile women (Figures 1a and b). The percentage of CD4^+^Foxp3^+^ Tregs in the peritoneal fluid from women with endometriosis was furthermore highest at stage III–IV (Figures 1a and b).

A significant increase in IL-10 levels was also observed in peritoneal fluid from women with endometriosis of stage III–IV (Figure 1c) while there was no statistical difference in IL-10 concentration between healthy controls and women with endometriosis at stage I–II (Figure 1c). The concentration of transforming growth factor-*β* (TGF-*β*), another key cytokine involved in CD4^+^Foxp3^+^ Treg function, was lowest in the peritoneal fluid from healthy controls, elevated in women with early stage endometriosis and highest in women with advanced stages of endometriosis (Figure 1d).

We also found that the concentration of TECK in peritoneal fluid increased with the progression of endometriosis (Figure 1e). A similar expression pattern was observed in primary ESCs, as TECK expression increased progressively from ESCs from healthy women to eutopic and ectopic ESCs from women with endometriosis (Figure 1f). Next, we established a co-culture model with ESCs and U937 cells (a macrophage cell line) to imitate the abdominal microenvironment in endometriosis. Co-culture led to a marked increase in TECK concentration compared with ESCs alone or U937 cells alone (Figure 1g).

These results suggest that there is a positive correlation between Tregs, IL-10 and TGF-*β* concentration, TECK concentration in peritoneal fluid and endometriosis progression. Statistical analysis revealed a strong positive correlation between the percentage of Tregs and TECK concentration with the development of endometriosis, suggesting that the enhanced production of TECK may have induced Treg differentiation in the peritoneal fluid of women with endometriosis (*R*^2^=0.9681, Supplementary Figure 1).

### ESC- and macrophage-derived TECK promotes Treg differentiation and IL-10 and TGF-*β* production by activating the AKT/STAT3 signaling pathway

**Figure 2:**
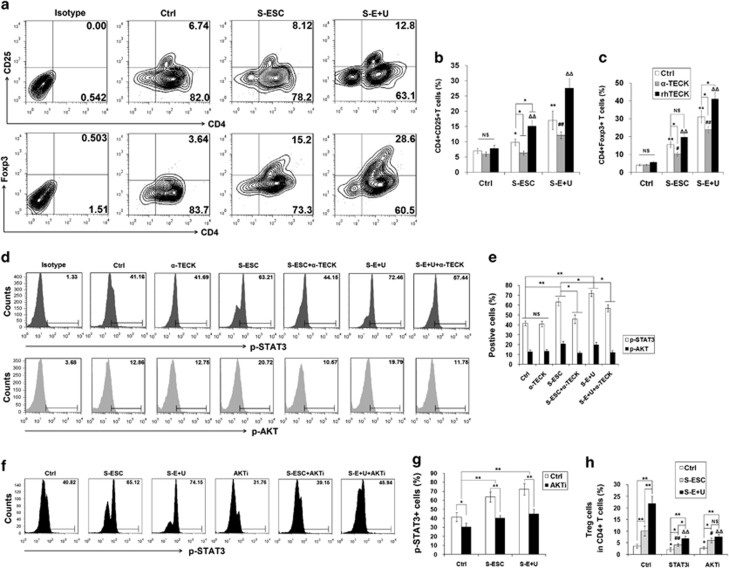
TECK stimulates Treg differentiation. After activation with anti-CD3 and anti-CD28 antibodies for 48 h, naïve T cells were cultured with S-ESC or S-E+U in the presence or absence of anti-human TECK neutralizing Abs (*α*-TECK) (5 *μ*g/ml) or recombinant human TECK (rhTECK) (100 ng/ml) (**a**–**c**) for 5 days and an AKT or STAT3 inhibitor (**d**–**h**) for another 24 h. The number of CD4^+^CD25^+^ and CD4^+^Foxp3^+^ Treg cells in the total CD4^+^ T cell population was determined by flow cytometry (**a**–**c**, **h**). The phosphorylation of STAT3 and AKT in naïve T cells was analyzed by flow cytometry after incubation with S-ESC, S-E+U and/or *α*-TECK, an AKT inhibitor for 24 h (**d**–**g**). S-ESC: supernatant from ESCs isolated from ectopic lesions; S-U937: supernatant from U937 cells; S-E+U: supernatant from co-cultured ESCs and U937 cells. Data are expressed as the mean±S.D. ^#^*P*<0.05, ^##^*P*<0.01 compared with vehicle control; ^#^*P*<0.05, ^##^*P*<0.01 compared with the S-ESC group; ^Δ^*P*<0.05, ^ΔΔ^*P*<0.01 compared with the S-E+U group

To define the relationship between TECK expression and Treg differentiation, we treated naïve T cells from peripheral blood from healthy women with the supernatant from ESCs (S-ESC) from ectopic lesions or the supernatant from co-cultured ESCs and U937 cells (S-E+U), with or without anti-human TECK neutralizing Abs (*α*-TECK) or recombinant human TECK (rhTECK). As shown in Figure 2, S-ESC promoted the differentiation of naïve T cells to CD4^+^CD25^+^ T cells and CD4^+^Foxp3^+^ Tregs (Figures 2a–c). S-E+U further enhanced the stimulatory effect on Treg differentiation (Figures 2a–c). These effects were significantly inhibited by *α*-TECK but enhanced by rhTECK (Figures 2b and c).

Further investigation of the down-stream signaling pathways showed that S-ESC and S-E+U activated STAT3 and AKT signaling in CD4^+^ T cells, and blocking TECK abolished these effects (Figures 2d and e). However, TECK did not regulate other signaling molecules (P38, ERK1/2, NF-*κ*B p65, STAT4 and STAT5) (Supplementary Figure 2). Blocking AKT signaling with an AKT inhibitor reversed the stimulatory effect of S-ESC and S-E+U on STAT3 phosphorylation (Figures 2f and g), and treatment with a STAT3 or AKT inhibitor led to a significant decrease in the induction of Treg differentiation by S-ESC and S-E+U (Figure 2h). These results indicate that the Treg differentiation induced by ESC- and macrophage-derived TECK in the peritoneal fluid is mainly dependent on the AKT/STAT3 signaling pathway.

**Figure 3:**
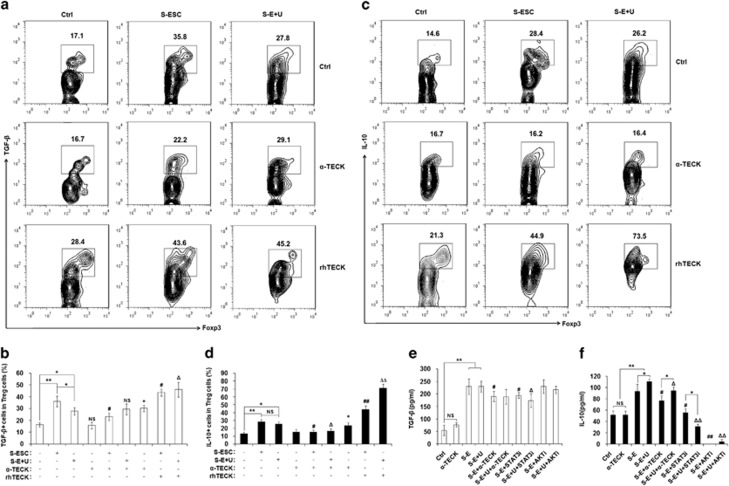
TECK promotes IL-10 and TGF-*β* in Tregs. After activation with anti-CD3 and anti-CD28 antibodies for 48 h, naïve T cells were cultured with S-ESC or S-E+U in the presence or absence of anti-human TECK neutralizing Abs (*α*-TECK) (5 *μ*g/ml) or recombinant human TECK (rhTECK) (100 ng/ml) for 5 days, then further incubated with ionomycin (100 ng/ml), PMA (100 ng/ml) and BFA (10 *μ*g/ml) for 4 h. The levels of intracellular TGF-*β* (**a**, **b**) and IL-10 (**c**, **d**) in CD4^+^Foxp3^+^Tregs were analyzed by flow cytometry. CD4^+^CD25^+^ regulatory T cells were isolated from the peripheral blood of healthy fertile women by MACS and incubated with S-ESC, S-E+U, anti-TECK neutralizing Abs, STAT3 (10 *μ*M) and/or AKT inhibitors (30 *μ*M). The amount of TGF-*β* (**e**) and IL-10 (**f**) secreted by the Tregs into the supernatant was determined. Data are expressed as the mean±S.D. *P<0.05 or **P<0.01 compared with medium/vehicle control; ^#^*P*<0.05, ^##^*P*<0.01 compared with S-ESC group without *α*-TECK and rhTECK; ^Δ^*P*<0.05, ^ΔΔ^*P*<0.01 compared with the S-E+U group without *α*-TECK and rhTECK

To identify whether TECK signaling is involved in the regulation of IL-10 and TGF-*β* production by Tregs, we isolated CD4^+^CD25^+^ regulatory T cells (Tregs) from peripheral blood from healthy fertile women using magnetic affinity cell sorting (MACS), and treated them with S-ESC, S-E+U, STAT3 and or AKT inhibitors. As shown in Figure 3, both S-ESC and S-E+U stimulated the production and secretion of TGF-*β* and IL-10 by Tregs (Figures 3a–f). Moreover, treatment with the anti-*α*-TECK and the STAT3 inhibitor decreased the secretion of TGF-*β* and IL-10 by Tregs (Figures 3e and f). Interestingly, the AKT inhibitor strongly inhibited IL-10 secretion but had no significant effect on TGF-*β* release (Figures 3e and f). Our observations suggest that TECK derived from ESCs and macrophages promote Treg differentiation and TGF-*β* and IL-10 production by activating the AKT/STAT3 signaling pathway.

### TECK reduces Treg apoptosis by downregulating Fas and FasL expression

**Figure 4:**
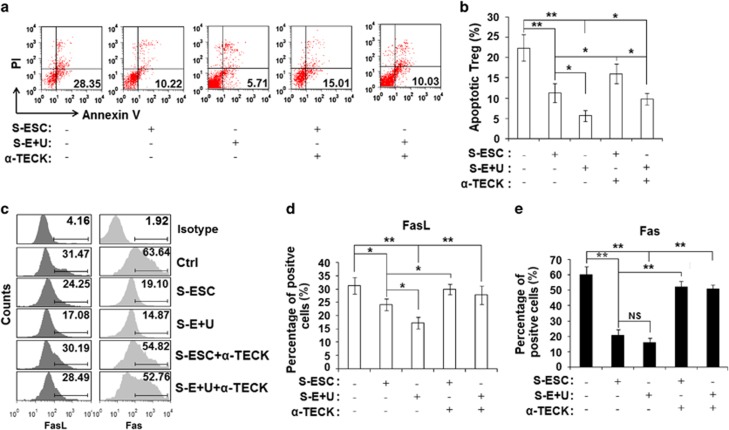
TECK represses Treg apoptosis. CD4^+^CD25^+^ Tregs were isolated from the peripheral blood of healthy women by MACS and cultured in S-ESC or S-E+U with or without *α*-TECK for 48 h. The Tregs were then stained for the Annexin V-FITC assay (**a, b**), and the percentages of FasL^+^ (**c, d**) and Fas^+^ Treg cells (**c, e**) were determined. Data are expressed as the mean±S.D.

To further test the influence of TECK on Treg function, we isolated CD4^+^CD25^+^ Tregs from the peripheral blood of healthy fertile women using MACS and then examined Treg apoptosis in response to TECK. As shown in Figure 4, treatment with S-ESC significantly repressed Treg apoptosis, and treatment with S-E+U further decreased Treg apoptosis to the lowest levels we observed (Figures 4a and b). However, treatment with *α*-TECK partly restored the level of Treg apoptosis induced by S-ESC and S-E+U (Figures 4a and b).

Further analysis showed that S-ESC and S-E+U downregulated expression of the apoptosis-related molecules Fas ligand (FasL) and Fas levels in Tregs and that these inhibitory effects were abrogated by treatment with *α*-TECK (Figures 4c–e). Stimulation with recombinant human soluble FasL protein partly abolished the protective effect of S-ESCs and S-E+U on Tregs (Supplementary Figures 3A and B). In contrast, blocking the FasL/Fas interaction with recombinant human soluble Fas (sFas) protein further repressed Treg apoptosis in the S-E and S+E+U cultures (Supplementary Figures 3A and B). These results suggest that TECK from ESCs and macrophages restricts Treg apoptosis in the peritoneal fluid of women with endometriosis by downregulating the expression of FasL and Fas.

### TECK enhances the CD4^+^CD25^+^ Tregs-mediated suppression of CD4^+^CD25^−^ effector T cells

**Figure 5:**
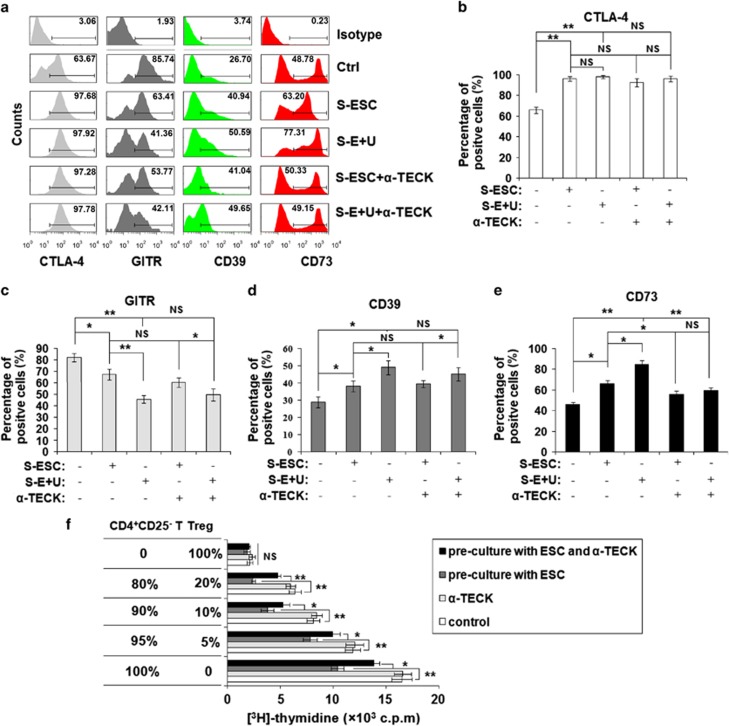
TECK enhances the suppression of CD4^+^CD25^−^ effector T cell by CD4^+^CD25^+^ regulatory T cells. (**a**–**e**) We quantified the number of CTLA-4, GTIR, CD73 and CD39-positive CD4^+^CD25^+^ Tregs after treatment with S-ESC or S-E+U and or *α*-TECK for 48 h. (**f**) After co-culture with ESCs (1 × 10^5^ cells/well) in the presence or absence of *α*-TECK for 48 h, CD4^+^CD25^+^Tregs were co-cultured with CD4^+^CD25^−^T cells at different ratios (2 × 10^5^ cells/well at the following ratios: 0%/100%, 80%/20%, 90%/10%, 95%/5% and 100%/0% CD4^+^CD25^−^ T/CD4^+^CD25^+^Tregs). Incorporated [^3^H] thymidine was added to evaluate the cell proliferation. Data are expressed as the mean±S.D.

To determine whether TECK regulates the suppressive ability of Tregs, we examined the expression of the surface molecules CTLA-4, GTIR, CD39 and CD73 on Tregs. We found that both S-ESC and S-E+U regulated the levels of CTLA-4, GTIR, CD39 and CD73 expression (Figures 5a–e). However, treatment with *α*-TECK only led to a decrease in CD73 expression by Tregs induced with S-ESC or S-E+U (Figures 5a and e).

Analysis of cell proliferation by incorporated [^3^H] thymidine showed that ESCs from ectopic lesions significantly enhanced the CD4^+^CD25^+^ Treg-mediated suppression of CD4^+^CD25^−^ effector T-cell proliferation in a number-dependent manner (Figure 5f). Treatment with *α*-TECK partially reversed the effects induced by the eutopic ESCs (Figure 5f). Taken together, these results suggest that TECK not only induces Treg differentiation but also enhances Treg function.

### Tregs stimulate ESC proliferation and invasion by secreting IL-10 and TGF-*β*

**Figure 6:**
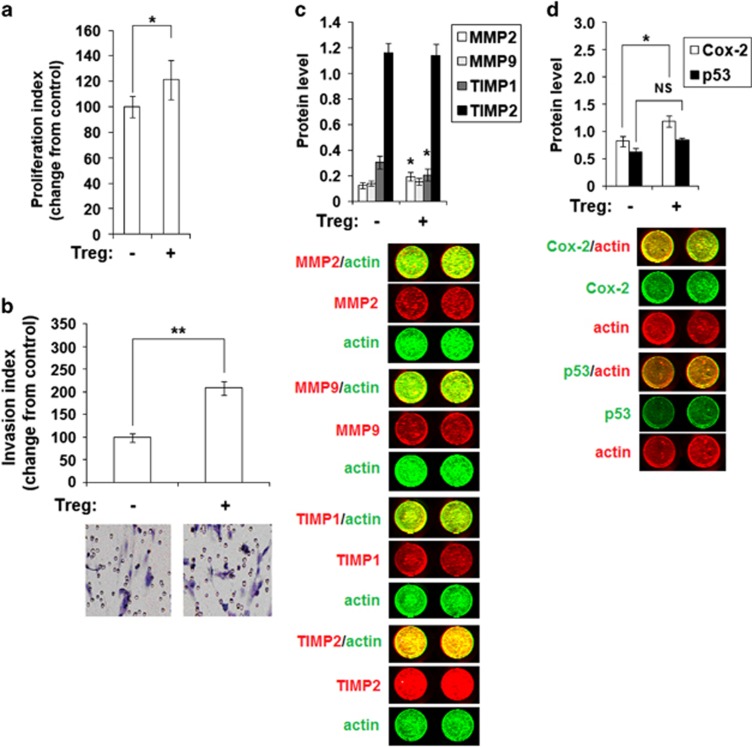
Tregs enhance ESC proliferation and invasiveness. (**a**) ESCs from ectopic lesions from patients with endometriosis were co-cultured with or without CD4^+^CD25^+^Tregs for 48 h. BrdU proliferation (**a**), Matrigel invasion (**b**) and In-cell Western assays (**c**, **d**) were performed to examine the proliferation, invasiveness and MMP2/9, TIMP1/2, Cox-2 and p53 expression in ESCs, respectively. MMP2, MMP9, TIMP1 and TIMP1 (red), actin (green); Cox-2 and p53 (green), actin (red). Original magnification: × 200 (**b**). All data are expressed as the mean±S.D. *P<0.05 compared with the vehicle control

To further investigate the impact of Treg on ESC behavior, we co-cultured ESCs from ectopic lesions with Tregs isolated from the peripheral blood of healthy women, and found that Tregs promoted ESC proliferation and invasiveness (Figures 6a and b). In-cell Western analysis showed that Tregs only modestly increased the expression of the invasion-related molecules MMP2 and cyclo-oxygen-ase-2 (Cox-2). Tregs decreased expression of tissue inhibitor of metalloproteinases 1 (TIMP1) but did not influence the expression of MMP9, TIMP2 and p53 in ESCs (Figures 6c and d).

**Figure 7:**
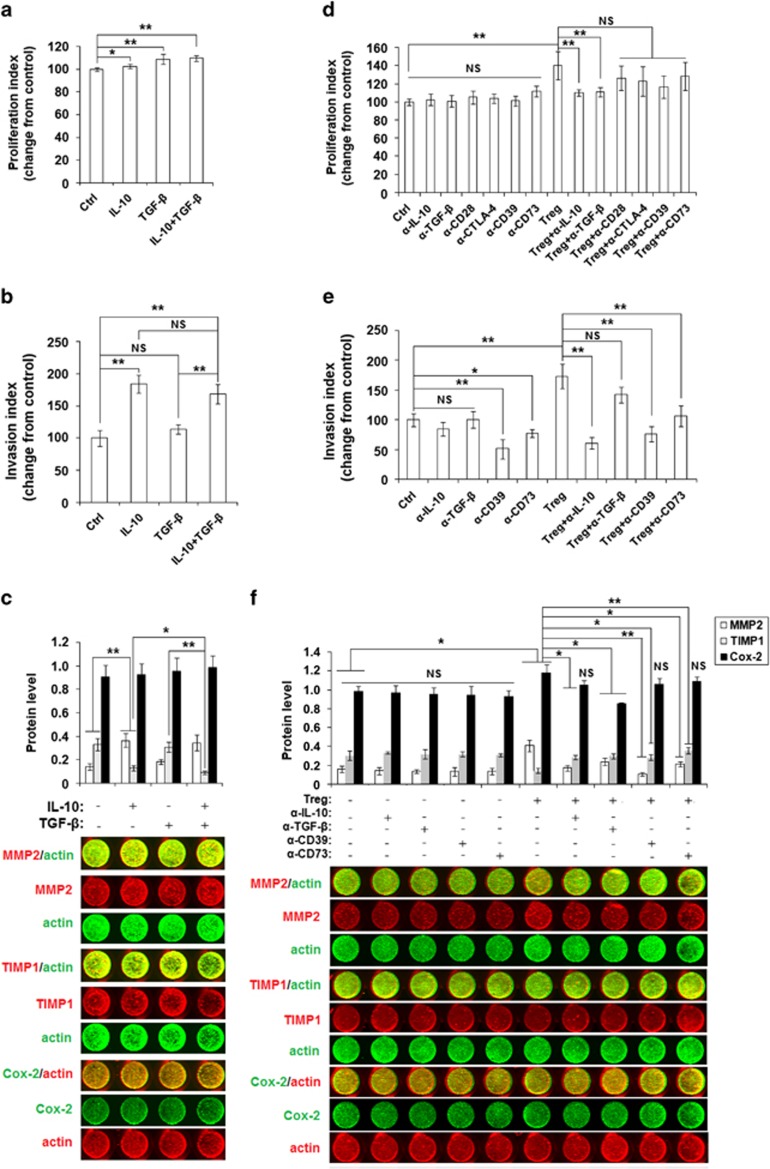
Tregs stimulation of ESC proliferation and invasiveness is dependent on IL-10 and TGF-*β*. (**a**) We treated ESCs isolated from eutopic endometrium from patients with endometriosis with rhIL-10 and/or rhTGF-*β* for 48 h and analyzed the proliferation (**a**), invasion (**b**) and the expression of MMP2, TIMP1 and Cox-2 (**c**). In addition, ESCs were co-cultured with CD4^+^CD25^+^ Tregs and/or anti-human IL-10, TGF-*β*, CD28, CTLA-4, CD39, CD73 neutralizing antibodies for 48 h. BrdU proliferation (**d**), Matrigel invasion (**e**) and In-cell Western assays (**f**) were performed to examine the proliferation, invasiveness, and MMP2, TIMP1 and Cox-2 expression in ESCs, respectively. MMP2 and TIMP1 (red), actin (green); Cox-2 (green), actin (red). All data are expressed as the mean±S.D.

To test whether two key cytokines are involved in regulating ESC behavior, we treated ESCs from ectopic lesions with two Treg cytokines and found that recombinant human IL-10 protein (rhIL-10) promoted ESC proliferation (Supplementary Figure 4 and Figure 7a) and invasiveness (Figure 7b) in a dose-dependent manner. However, rhTGF-*β* enhanced ESC proliferation, but not invasiveness (Supplementary Figure 4 and Figures 7a and b). Unlike rhTGF-*β*, treatment with rhIL-10 increased MMP2 and decreased TIMP1 expression (Figure 7c). In addition, stimulation with a combination of rhIL-10 and rhTGF-*β* strengthened the inhibition of TIMP1 expression by ESCs (Figure 7c).

To determine the mechanism of the Treg effects on ESC behaviors, we incubated ESCs with Tregs and/or anti-human IL-10, TGF-*β*, CD28, CTLA-4, CD39, CD73 neutralizing Abs. As shown, only anti-human IL-10 and TGF-*β* neutralizing antibodies (Abs) partly abrogated the effect of Tregs on ESC proliferation (Figure 7d). Blocking CD39 or CD73 with neutralizing Abs directly restricted ESC invasion (Figure 7e). Blocking IL-10, CD39 or CD73 decreased Treg stimulation of ESC invasion (Figure 7e). Treatment with anti-human IL-10, TGF-*β*, CD39 or CD73 neutralizing Abs also restored the expression of MMP2 and TIMP1 in ESCs induced by Tregs (Figure 7f).

These data indicate that Tregs increase the expression of MMP2, decrease the expression of TIMP1 by ESCs, and further feedback stimulate ESC growth and invasiveness in the endometriotic milieu by functional molecules IL-10, TGF-*β* and CD73.

### TECK promotes Treg differentiation and function and accelerates the growth of endometriosis lesions in mice

**Figure 8:**
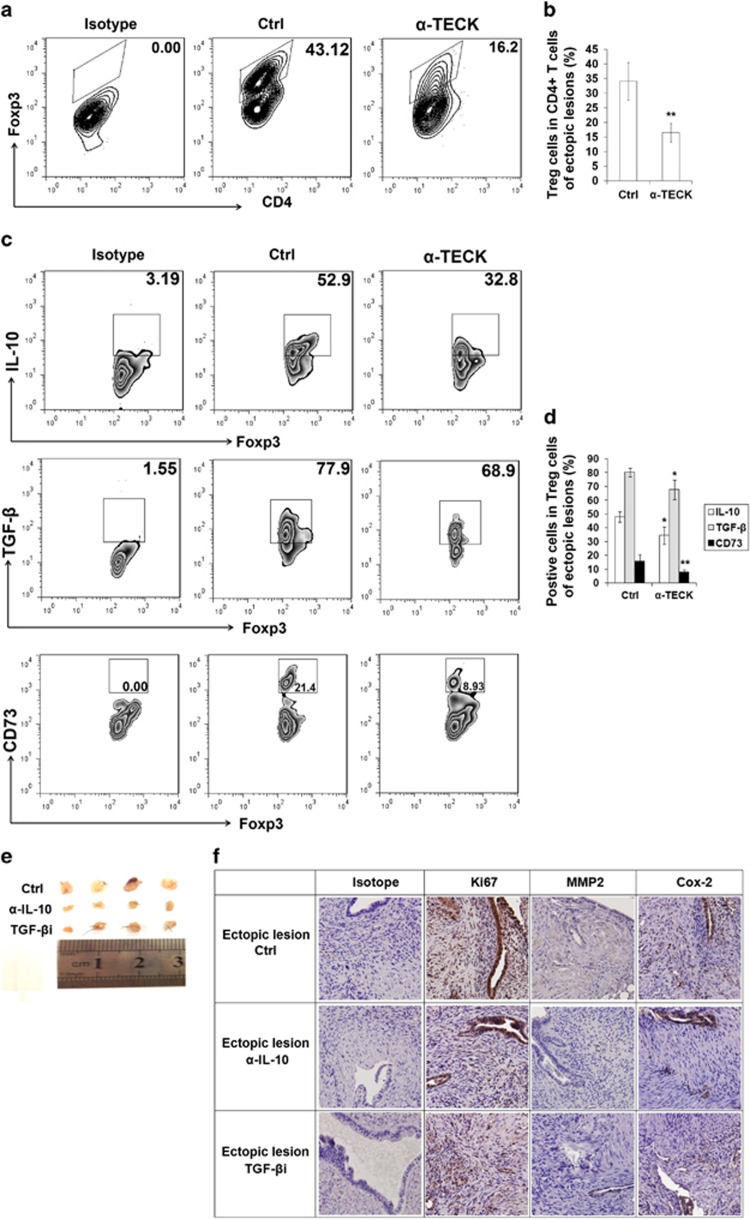
TECK promotes Treg differentiation and function and accelerates the growth of endometriotic lesions in mice. Endometriosis-like lesions were induced in C57B/L6 mice by transplanting uterine tissues samples. The mice were treated with anti-mouse TECK neutralizing Abs (*α*-TECK) (50 *μ*g/mice) (**a**–**d**), anti-mouse IL-10 neutralizing Abs (50 *μ*g/mouse) or TGF-*β* receptor antagonist SB431542 (10 *μ*M/mouse) (**e**, **f**) by intraperitoneal injection every week after surgery. Vehicle-only injection was used as the control. After 2 weeks, the endometriosis-like lesions in mice were removed and digested, and the percentage of CD4^+^Foxp3^+^ Tregs (out of the total number of CD4^+^ T cells) (**a, b**) and the level of IL-10, TGF-*β* and CD73 expression in Tregs were determined (**c**–**d**) by flow cytometry. Administration of anti-mouse IL-10 neutralizing Abs (*α*-IL-10) or TGF-*β* receptor antagonist (SB431542) (*α*-TGF-*β*) limited endometriosis lesion growth (**e**). The expression of Ki67, MMP2 and Cox-2 in mouse ESCs was evaluated by immunohistochemistry (**f**). Original magnification: × 200. All data are expressed as the mean±S.D. *P<0.05, **P<0.01 compared with the vehicle control

To determine whether TECK is involved in Treg differentiation and the development of endometriosis *in vivo*, we used the C57B/L6 mouse i.p. endometriosis model (Supplementary Figure 5A). Intraperitoneal injection of anti-mouse *α*-TECK significantly slowed endometriosis lesion growth (Supplementary Figure 5B). Consistent with our *in vitro* results, blocking TECK resulted in a marked decrease in Treg differentiation (Figures 8a and b), and decreased the expression of IL-10, TGF-*β* and CD73 by Tregs in endometriosis lesions (Figures 8c and d). Administration with anti-mouse IL-10 neutralizing Abs or TGF-*β* receptor antagonist (SB431542) also limited the growth of endometriosis lesions (Figure 8e) and downregulated the expression of Ki67, MMP2 and Cox-2 by ESCs and endometrial glandular epithelial cells (EECs) in the endometriosis lesions (Figure 8f). These results are similar to the results obtained by blocking TECK (Supplementary Figures 5B and 6).

Collectively, these results suggest that TECK promotes Treg differentiation in the endometriotic milieu and that the Tregs, in turn, stimulate the development of endometriosis through IL-10, TGF-*β* and CD73.

## Discussion

Endometriosis results from increased cellular proliferation, adhesion and invasion of the retrograde endometrium in response to appropriate stimuli. The retrograded endometrial fragments into the peritoneal cavity trigger a suboptimal immune response that does not adequately clear the implanted tissues, resulting in their continued survival and growth and thus the development of endometriosis.

CD4^+^CD25^+^Foxp3^+^ regulatory cells are a component of the immune system that suppresses immune responses of other cells. This is an important ‘self-check' built into the immune system to prevent excessive reactions.^15^ Many signaling molecules are involved in regulating Tregs, such as phosphatidyl inositol 3-kinase.^16^ Podgaec *et al.*^7^ and Olkowska-Truchanowicz *et al.*^8^ reported that the percentage of Tregs is increased in peritoneal fluid. These studies suggest that the abnormally high levels of Tregs may be involved in the endometriosis-related immune tolerance. However, the mechanism of Treg differentiation and the role of Treg-ESC crosstalk in this process were not well-understood.

TECK has a key role in the segregation and compartmentalization of the mucosal immune system through recruitment of immune cells to specific locations.^17^ In our previous study we showed that TECK derived from endometriotic-associated cells enhances ESC invasion by upregulating the expression of MMP2/9.^14^ In this study, we further show that the percentage of CD4^+^Foxp3^+^ T cells in peritoneal fluid is positively correlated with the progression of endometriosis. The concentration of IL-10, TGF-*β* and TECK in peritoneal fluid was also consistent with the number of CD4^+^Foxp3^+^ T cells. In addition, the secretion of TECK by ESCs from ectopic lesions was higher than that from eutopic endometrium with or without endometriosis. Co-culturing ESC with a macrophage cell line, U937, significantly increased the levels of TECK production. Therefore, we hypothesized that TECK expression by ESCs and macrophages may regulate Treg differentiation and development, further reinforcing the dialogue between ESCs and Tregs that is involved in the development of endometriosis.

The key finding from our study is that the upregulation of TECK in endometriotic-associated cells (such as ESCs and macrophages) promotes Treg differentiation, IL-10 production and TGF-*β* production by activating the AKT/STAT3 signaling pathways in T cells, but decreases FasL and Fas expression and further inhibits Treg apoptosis possibly by downregulating ‘suicide' and ‘homicide'. The upregulation of TECK also increases CD73 expression and enhances the suppressive effect of Tregs on the CD4^+^CD25^−^ effector T cells. In turn, these educated Tregs promote the expression of MMP2 and Cox-2 and inhibit the expression of TIMP1, and stimulate ESC proliferation and invasion in IL-10, TGF-*β* and CD73-dependent or -independent manners. The integral effects of these signaling molecules create an immune-tolerant microenvironment that promotes ESC survival and invasion, which leads to the development of endometriosis (Supplementary Figure 7).

Following cell contact, Tregs may kill responder T cells by a granzyme-dependent or perforin-dependent mechanism.^18,19^ Alternatively, the Tregs may deliver a negative signal to responder T cells via one of the following mechanisms: upregulating intracellular cyclic AMP, which leads to the inhibition of T-cell proliferation and IL-2 expression;^20^ generating pericellular adenosine catalyzed by CD39 (ectonucleoside triphosphate diphosphohydrolase 1) and CD73 (ecto-5′-nucleotidase) expression by Tregs;^21, 22, 23^ or interacting with B7 (CD80 and CD86) expressed by responder T cells.^24^ It is well-known that the FasL/Fas-mediated cell death pathway represents typical apoptotic signaling in many cell types.^25,26^ According to our results, TECK from ESCs and macrophages downregulated the expression of FasL/Fas and reduced the apoptosis of Tregs. Furthermore, the supernatant from co-cultured ESCs and macrophages modulated the expression of CTLA-4, GITR, CD39 and CD73 by Tregs. However, TECK only was involved in regulating Treg expression of CD73 triggered by ESCs and macrophages, and further enhancing the suppressive effect of Tregs on CD4^+^CD25^−^T cells. Thus, these TECK-educated Tregs may have a stronger coordinated ability to assist ESC immune escape of ESCs in the pelvic region by creating a local immune-tolerant microenvironment. In addition, our results indicate that, in addition to TECK, other molecules from ESCs and macrophages also play an important role in regulation of CTLA-4, GITR and CD39 expression by Tregs. However, more research is needed to determine the molecular mechanisms of this regulation.

The immunosuppressive cytokines TGF-*β* and IL-10 have been implicated in endometriosis. Tagashira *et al.*^27^ reported that IL-10 attenuates TNF-*α*-induced IL-6 synthesis via the NF-*κ*B and MAPK pathways in endometriotic stromal cells, which suggests that increased IL-10 expression may have a significant role in regulating the balance between complex pro- and anti-inflammatory behaviors in endometriosis. TGF-*β* has been implicated in gene expression, cell motility, proliferation, apoptosis, differentiation, immune responses and tumorigenesis.^28,29^ TGF-*β*s are abundantly and differentially expressed in the endometrium and are also secreted by endometrial stroma, glands and macrophages into the uterine fluid, which suggest that they may participate in scarless postmenstrual regeneration of endometrium.^30^ These findings echoed our results to a certain degree. The increased levels of IL-10 and TGF-*β* produced by Tregs in response to TECK and other cell types (such as ESCs and macrophages) may further modulate the progression of endometriosis through anti-inflammatory pathways and directly enhance the biological behavior of ESCs in the peritoneal cavity.

The initial phase of endometriosis is an invasion event that requires ECM breakdown and tissues repair, which requires the increased activity of MMP-1, MMP-2 and MMP-9.^31^ Indeed, MMPs and TIMPs levels have been correlated with the development and progression of endometriosis.^31,32^ Prostaglandins (PGs) are bioactive lipids produced from arachidonic acid by cyclooxygenase (COX) enzymes and a specific terminal prostanoid synthetase enzyme. PGE2 has an important role in the pathogenesis of endometriosis. COX-2 has also been shown to regulate the survival, migration and invasion of endometriotic cells.^32,33^ In this study, we found that Tregs upregulated the expression of MMP2 and COX-2 and downregulated TIMP1 expression, but did not influence the levels of MMP9, TIMP2 and p53. These effects were dependent on the functional molecules IL-10, TGF-*β* and CD73. Unlike IL-10, TGF-*β* was mainly involved in the regulation of ESC proliferation. Our *in vivo* experiments also confirmed that TECK promotes Treg differentiation. Similar to neutralizing TECK, blocking IL-10 or TGF-*β* led to a significant decrease in Ki67, MMP2 and Cox-2 expression by ESCs and EECs, and a marked reduction in ectopic lesions in mice.

In summary, our results suggest that abnormally high TECK secretion by ESCs may initiate local immune tolerance in the ectopic milieu by upregulating the quantity and function of Tregs. The increase in Tregs could lead to the difficulty in clearing ESCs due to the stimulation of ESC growth and invasion into the peritoneal cavity. The excessive growth and deep infiltration of ESCs further promote TECK secretion and Treg differentiation, leading to a vicious cycle that promotes the development of endometriosis. Taken together, our findings help to elucidate the crosstalk between ESC and Treg in the pathogenesis of endometriosis.

## Materials and Methods

### Antibodies

Fluorescein isothiocyanate (FITC)-conjugated anti-human CD4, Phycoerythrin (PE)-conjugated anti-human Foxp3, Allophycocyanin (APC)-conjugated anti-human IL-10, Percp5.5-conjugated anti-human TGF-*β*, PE-conjugated anti-human Fas, APC-conjugated anti-human FasL, FITC-conjugated anti-human CTLA-4, PE-conjugated anti-human glucocorticoid-induced tumor necrosis factor receptor (GITR), Percp5.5-conjugated anti-human CD39, APC-conjugated anti-human CD73, Percp5.5-conjugated anti-mouse CD4, PE-conjugated anti-mouse CD25, FITC-conjugated anti-mouse Foxp3, PE-conjugated anti-mouse CD73, Percp5.5-conjugated anti-mouse TGF-*β*, APC-conjugated anti-mouse IL-10 and isotypic IgG Abs were purchased from Biolegend (San Diego, CA, USA); BD Phosflow PE-conjugated anti-human p-STAT3, Alex Flour 647-conjugated anti-human p-AKT and isotype IgG Abs were purchased from BD Biosciences (San Jose, CA, USA); monoclonal and polyclonal Abs against human actin, Cox-2, p53, mouse Ki67, MMP2 and Cox-2 were purchased from Cell Signal Technology (Beverly, MA, USA); mouse anti-human MMP2/9, TIMP1/2 Abs were purchased from R&D Systems (Abingdon, UK); secondary IRDye 700DX-conjugated affinity purified (red fluorescence) anti-mouse, and IRDye 800DX-conjugated affinity purified (green fluorescence) anti-rabbit fluorescence Abs were purchased from Rockland, Inc (Gilbertsville, PA, USA); and anti-human TECK, IL-10, TGF-*β*, CD28, CTLA-4, CD39, CD73 and anti-mouse TECK and IL-10 neutralizing Abs were purchased from R&D Systems. The selective STAT3 inhibitor 5, 15-DPP, the AKT inhibitor LY294002 and the TGF-*β* receptor antagonist SB431542 were purchased from Sigma (Sigma-Aldrich Co. LLC, St. Louis, MO, USA).

### Patients

The protocol for this study was approved by the Institutional Review Board of Hospital of Obstetrics and Gynecology, Fudan University Shanghai Medical College, and written informed consent for participation was obtained from all participants. All of the eutopic endometrial and endometriotic tissues were obtained by laparoscopy from 49 patients with endometriosis (mean age 38.8 years; range 31–42) at the Hospital of Obstetrics and Gynecology, Fudan University Shanghai Medical College. The patients were classified according to the revised American Fertility Society (AFS) classification: 27 patients were in early stages (stage I and II), and 22 patients were in advanced stages (stage III and IV) stage. The patients had not received any GnRH analog or other hormonal drug in the 6 months prior to the surgical operation. All of the samples were obtained in the proliferative phase of the cycle, which was confirmed histologically according to established criteria. Normal endometrium was obtained from 11 disease-free women as healthy controls.

### Collection and preparation of peritoneal fluid

Peritoneal fluid was aspirated from the *cul de sac* at the beginning of the standard laparoscopic procedure under general anesthesia. Samples of peritoneal fluid contaminated by blood were excluded from the study. The samples were centrifuged at 1500 r.p.m. for 10 min, and then frozen at −80 °C. The IL-10, TGF-*β* and TECK concentrations were determined using ELISA kits (Shanghai ExCell Biology, Inc, Shanghai, China) according to the manufacturer's instructions.

The mononuclear cells were isolated from the peritoneal fluid using lymphocyte separation medium (Dakewe Biotech Co., Ltd., Shenzhen, China) and density-gradient centrifugation at 2000 r.p.m. for 20 min at 4 °C. The cells were collected, washed in ice-cold PBS and suspended in Ab staining buffer at the desired concentration for flow cytometry.

### Cell culture

The endometrial tissues (from women with ovarian and pelvic endometriomas and healthy controls) and endometriotic ovarian lesion tissues were collected under sterile conditions and transported to the laboratory on ice in Dulbecco's modified Eagle's medium (DMEM)/F-12 (Gibco, Grand Island, NY, USA) supplemented with 10% fetal calf serum (FCS; Hyclone, Logan, UT, USA). The ESCs were isolated according to previously described methods.^14,34^ Immunocytochemistry showed >95% vimentin-positive and cytokeratin-negative ESCs. The human monocytic cell line U937 (purchased from the Bank of Cell, the Chinese Academy of Sciences, Shanghai, China) was maintained in DMEM/F-12 supplemented with 10% FCS.

### ELISA

To evaluate the level of secreted TECK, ESCs from healthy endometrium, eutopic endometrium and ectopic lesions from women with endometriosis were cultured in 24-well plates for 48 h (1 × 10^5^ cells/well) (*n*=6). In addition, ESCs (1 × 10^5^ cells/well) from eutopic endometrium from women with endometriosis were cultured with U937 cells (1 × 10^5^ cells/well) for 48 h. ESCs alone and U937 cells alone were included as controls. The cell culture supernatant was then harvested, centrifuged to remove cellular debris and stored at −80 °C. The ESC supernatant was assayed by ELISA according to the manufacturer's instructions, to detect the levels of secreted TECK.

CD4^+^CD25^+^ Tregs were incubated with culture medium only, the S-ESC or S-E+U, plus an AKT inhibitor (LY294002, 30 *μ*M) or a STAT3 inhibitor (5, 15-DPP, 10 *μ*M)), vehicle was added as the control. After 3 days of culture, the supernatant from the CD4^+^CD25^+^ Tregs was collected to determine the secretion levels of IL-10 and TGF-*β*.

### Isolation of naïve CD4^+^ T cells, CD4^+^CD25^+^ Tregs and CD4^+^CD25^−^ T cells

PBMCs were isolated from healthy fertile women as described previously.^35^ Naïve CD4^+^ T cells, CD4^+^CD25^+^ Tregs and CD4^+^CD25^−^ T cells were purified by MACS using the human naïve CD4^+^ T Cell Isolation Kit and the human CD4^+^CD25^+^ Treg Isolation Kit, respectively, according to the manufacturer's instructions (Miltenyi Biotec GmbH, Bergisch Gladbach, Germany). The purity of the separated naïve CD4^+^ T cells, CD4^+^CD25^+^ T cells and CD4^+^CD25^−^ T cells was >95%, as determined by flow cytometry.

### Treatment of naïve CD4^+^ T cells and CD4^+^CD25^+^ Tregs

The isolated naïve CD4^+^ T cells or CD4^+^CD25^+^ Tregs were activated with 5 *μ*g/ml anti-CD3, 2 *μ*g/ml anti-CD28 (eBioscience, San Diego, CA, USA) for 2 days. The cells were then collected, washed and incubated with culture medium only, the S-ESC from ectopic lesions or S-E+U cells, plus anti-human *α*-TECK (5 *μ*g/ml), rhTECK (100 ng/ml), an AKT inhibitor (LY294002) or a STAT3 inhibitor (5, 15-DPP), vehicle was added as a control. After 5 days of culture, the naïve CD4^+^ T cells and CD4^+^CD25^+^ Tregs were collected for flow cytometry.

### Flow cytometry

To identify and evaluate the Tregs, mononuclear cells from peritoneal fluid were stained with an anti-CD4 monoclonal Ab, followed by intracellular staining of Foxp3 according to the manufacturer's instructions. Flow cytometry was performed to analyze the percentage of CD4^+^Foxp3^+^ T cells, IL-10 and TGF-*β* levels, phosphorylation of STAT3 and AKT in naïve CD4^+^ T cells and the expression of Fas, FasL, CTLA-4, GITR, CD39 and CD73 in CD4^+^CD25^+^ Tregs, using isotypic IgG Abs as controls.

The samples were analyzed using a FACS Calibur flow cytometer (Becton Dickinson, Franklin Lakes, NJ, USA) and Cellquest software (Becton Dickinson). The statistical analysis was conducted using isotype-matched controls as references.

### Annexin V-FITC assay for apoptosis

The level of apoptosis in CD4^+^CD25^+^ Tregs was measured by an apoptosis assay. Phosphatidylserine externalization was quantified by flow cytometry using a commercially available annexin V-FITC apoptosis detection kit (Invitrogen, Carlsbad, CA, USA), as described previously.^34^

### Suppression of CD4^+^CD25^−^ effector T-cell proliferation by CD4^+^CD25^+^ Tregs

After co-culture with or without ESCs and/or anti-human *α*-TECK, the CD4^+^CD25^+^ Tregs were collected and further incubated with CD4^+^CD25^−^T cells. The CD4^+^CD25^−^T cells and CD4^+^CD25^+^ Tregs were combined at ratios from 100%/0% to 0%/100%. These cells were stimulated with 5 *μ*g/ml anti-CD3 mAb and 2 *μ*g/ml anti-CD28 mAb for 72 h. During the last 16 h, the cells were pulsed with 1 mCi of [^3^H] thymidine, then harvested on glass fiber filters and analyzed for incorporated [^3^H] thymidine in a beta-counter.

### Matrigel invasion, BrdU proliferation and In-cell Western assays

ESCs from ectopic lesions from women with endometriosis were co-cultured with recombinant human IL-10 (rhIL-10), rhTGF-*β*, CD4^+^CD25^+^ Tregs and/or anti-human IL-10, TGF-*β*, CD28, CTLA-4, CD39 and CD73 neutralizing Abs for 48 h, after which the cells were collected. We further analyzed ESC invasiveness, proliferation and expression of MMP2/9, TIMP1/2, Cox-2 and p53 by Matrigel invasion assay, BrdU cell proliferation assay (Millipore, USA) and In-cell Western assay, respectively, as described previously.^34^

For the In-cell Western assay, the expression level of the corresponding molecules was calculated as the ratio of the intensity of the target genes to actin.

### Intraperitoneal endometriosis model

Intraperitoneal endometriosis-like lesions were induced surgically by suturing uterine tissue samples to the abdominal wall, as described in Körbel *et al.*^36^ For autologous transplantation, the left uterine of the recipient animal was divided into four equal parts and sewn into four quadrants of the peritoneum. Endometriosis-like lesions developed along the transplanted uterine tissue samples. The mice were treated with anti-mouse *α*-TECK (50 *μ*g/mouse), anti-mouse IL-10 neutralizing Abs (50 *μ*g/mouse) or TGF-*β* receptor antagonist SB431542 (10 *μ*M/mice) every week after surgery. After 2 weeks, the endometriosis-like lesions were removed, minced on ice and digested with an enzyme mix containing Liberase and Dispase (Invitrogen). Then, we collected these cells and evaluated Treg differentiation and function by flow cytometry.

### Immunohistochemistry

Paraffin sections (5 *μ*m) of the endometriosis-like lesions and normal endometrium from mice were dehydrated in graded ethanol, then incubated with hydrogen peroxide and 1% bovine serum albumin/TBS to block endogenous peroxidase. The samples were then incubated with rabbit anti-mouse Ki67 Ab (15 *μ*g/ml), MMP2 Ab (25 *μ*g/ml), COX-2 Ab (25 *μ*g/ml) or rabbit IgG isotype overnight at 4 °C in a humid chamber. After washing three times with TBS, the sections were overlaid with peroxidase-conjugated goat anti-rabbit IgG (Golden Bridge International, Inc, Beijing, China), and the reaction was developed with 3,3-diaminobenzidine and counterstained with hematoxylin. The experiments were repeated five times.

### Statistics

All values are shown as the mean±S.D. The data were analyzed using a *t*-test or a one-way analysis of variance with least significant difference (equal variances assumed), or Tamhane's (equal variances not assumed) *post-hoc* test for multiple comparisons using the Statistical Package for the Social Sciences software version 16.0. Differences were considered statistically significant at *P*<0.05.

## Abbreviations

Ab: antibody
CCR9: CC chemokine receptor 9
TECK: thymus-expressed chemokine
Treg: Regulatory T cell
S-ESC: the supernatant from ESCs
S-E+U: the supernatant from co-cultured ESCs and U937 cells
*α*-TECK: anti-TECK neutralizing Abs
rhTECK: recombinant human TECK
MMP: Matrix metalloproteinase
COX: cyclooxygenase
