# Supplementary material for: CD4+Foxp3+ regulatory T cell differentiation mediated by endometrial stromal cell-derived TECK promotes the growth and invasion of endometriotic lesions
Source: Cell Death Dis. 2014 Oct 2;5(10):e1436–. doi: 10.1038/cddis.2014.414 (PMC4649519; doi:10.1038/cddis.2014.414)
Supplement: Supplementary Information [file cddis2014414x1.doc]

**Supplementary Information**

**Supplementary Methods**

**Reagents.** [BD Phosflow](http://www.bdbiosciences.com/nvCategory.jsp?action=SELECT&form=formTree_catBean&item=745719&wcm_page=/research/phosflow/productlist/&wcm_title=BD Phosflow  Product List)^TM^ Alex Flour 488-conjugated anti-human p-P38, Percp5.5-conjugated anti-human ERK1/2, Alex Flour 488-conjugated anti-human p-NF-κB, Percp5.5-conjugated anti-human STAT5, Alex Flour 647-conjugated anti-p-STAT4, and isotype IgG antibodies were purchased from BD Biosciences (USA); monoclonal and polyclonal antibodies against mouse Ki67, MMP2 and Cox-2 were purchased from Cell Signal Technology (Beverly, MA, USA); recombinant human soluble Fas Ligand (sFasL) protein and soluble Fas (sFas) protein were purchased from PeproTech Inc.; recombinant human IL-10, TGF-β proteins, anti-mouse TECK neutralizing antibody were purchased from R&D Systems; BrdU cell proliferation assay kit was purchased by Millipore, USA; and monoclonal and polyclonal antibodies against mouse Ki67, MMP2 and Cox-2 were purchased from Cell Signal Technology (Beverly, MA, USA).

**Flow cytometry.** Flow cytometry was performed to analyze the phosphorylation of P38, ERK1/2, NF-κB, STAT4, and STAT5 in naïve CD4^+^ T cells after treatment with S-ESC, S-E+U and or anti-TECK neutralizing antibody. The samples were analyzed using a FACS Calibur flow cytometer (Becton Dickinson, USA) and Cellquest software (Becton Dickinson). The statistical analysis was conducted by using isotype matched controls.

**Annexin V-FITC assay for apoptosis.** The level of apoptosis in CD4^+^CD25^+^ regulatory T cells was measured by an apoptosis assay. Phosphatidylserine externalization was quantified by flow cytometry using an annexin V-FITC apoptosis detection kit (Invitrogen, USA).

**BrdU proliferation assay.** ESCs from ectopic lesions from women with endometriosis were incubated with recombinant human IL-10 (rhIL-10) or rhTGF-β at different concentration for 48 h, and then the cells were collected. We further analyzed the proliferation of ESCs by BrdU cell proliferation assay.

**Immunohistochemistry.** Paraffin sections (5 um) of the endometriosis-like lesions and normal endometrium from mice were dehydrated, and incubated with hydrogen peroxide and 1% bovine serum albumin (BSA)/TBS to block endogenous peroxidase. The samples were then incubated with rabbit anti-mouse Ki67 Ab (15 ug/ml), MMP2 Ab (25 ug/ml), COX-2 Ab (25 ug/ml) or rabbit IgG isotype overnight at 4 °C in a humid chamber. After washing three times with TBS, the sections were overlaid with peroxidase-conjugated goat anti-rabbit IgG (Golden Bridge International, Inc, USA), and the reaction was developed with 3,3-diaminobenzidine (DAB) and counterstained with hematoxylin. The experiments were repeated five times.

**Supplementary Figure 1: A strong positive correlation between the percentage of Tregs and TECK concentration with the development of endometriosis.** The percentage of Tregs in the total CD4^+^ T cells and TECK concentration in peritoneal fluid from healthy controls and patients with endometriosis were analyzed by flow cytometry and ELISA assays, respectively. Then analysis of correlation showed that there was a strong positive correlation between the percentage of Tregs and TECK concentration in the peritoneal fluid (R^2^=0.9681).

**Supplementary Figure 2: TECK produced by ESCs and macrophage does not activate P38, ERK1/2, NF-κB, STAT5, and STAT4 signals.** Flow cytometry was performed to analyze the phosphorylation of P38, ERK1/2, NF-κB, STAT5, and STAT4 in naïve T cells, which treated with or without S-ESC, S-E+U and or anti-TECK neutralizing antibody for 24 h. All data are expressed as the mean±SD.

**Supplementary Figure 3: FasL/Fas interaction is involved in the effect of ESCs and macrophages on Treg apoptosis. (A, B)** The CD4^+^CD25^+^ regulatory T cells were isolated from peripheral blood from healthy fertile women using MACS and incubated with S-E or S-E+U, plus recombinant human FasL (rhFasL) or rhFas (**B**) for 48 h. The level of apoptosis in Tregs was analyzed. All data are expressed as the mean±SD. ^#^*P*<0.05, ^##^*P*<0.01 compared to medium/vehicle control; ^&&^*P*<0.01 compared to the S-ESC group; ^$$^*P*<0.01 compared to the S-E+U group. NS: no statistical difference.

**Supplementary Figure 4: Recombinant human IL-10 and TGF-β proteins promote ESC proliferation.** ESCs of ectopic lesions from women with endometriosis were incubated with recombinant human IL-10 (rhIL-10) (**A**) or rhTGF-β (**B**) at different concentrations for 48 h. Then ESC proliferation was determined by BrdU cell proliferation assay. All data are expressed as the mean±SD.

**Supplementary Figure 5: TECK stimulates endometriosis lesions growth.** (**A**) The C57B/L6 mice i.p. endometriosis model was constructed. For autologous transplantation, the left uterine of the recipient animal was divided into 4 equal parts, and sewn into four quadrants of the peritoneum. (**B**) Intraperitoneal injection of anti-mouse TECK neutralizing antibody (α-TECK) significantly slowed endometriosis lesions growth.

**Supplementary Figure 6:** **TECK promotes the expression of Ki67, MMP2 and COX-2 in mouse ESCs.** Intraperitoneal injection of anti-mouse TECK neutralizing antibody (α-TECK) every week, then the expression of Ki67, MMP2 and Cox-2 in ESCs from mouse endometriosis lesions was analyzed by immunohistochemistry. Original magnification: ×200.

**Supplementary Figure 7: Schematic roles of crosstalk between ESCs and regulatory T cells** **in the pathogenesis of endometriosis.** The up-regulation of TECK from ESCs and macrophages owing to inherent defects or the effect of macrophages regurgitate into peritoneal cavity, which promotes Treg differentiation by activating AKT and STAT3 signaling pathways, stimulates IL-10 and TGF-β production and CD73 expression, restricts Tregs apoptosis through decreasing the expression of Fas and FasL, and further enhances the suppressive effect on effective T cells. In turn, TECK-educated Tregs increase the expression of MMP2, TIMP1 and Cox-2, promotes ESC growth and invasion by IL-10 and TGF-β. The dialogue between ESC and Treg leads to the [vicious circle](http://dict.baidu.com/s?wd=a vicious circle) in the peritoneal cavity, which is involved in the pathogenesis of endometriosis.
