# Supplementary figures and images for: CD4+Foxp3+ regulatory T cell differentiation mediated by endometrial stromal cell-derived TECK promotes the growth and invasion of endometriotic lesions
Source: Cell Death Dis. 2014 Oct 2;5(10):e1436–. doi: 10.1038/cddis.2014.414 (PMC4649519; doi:10.1038/cddis.2014.414)

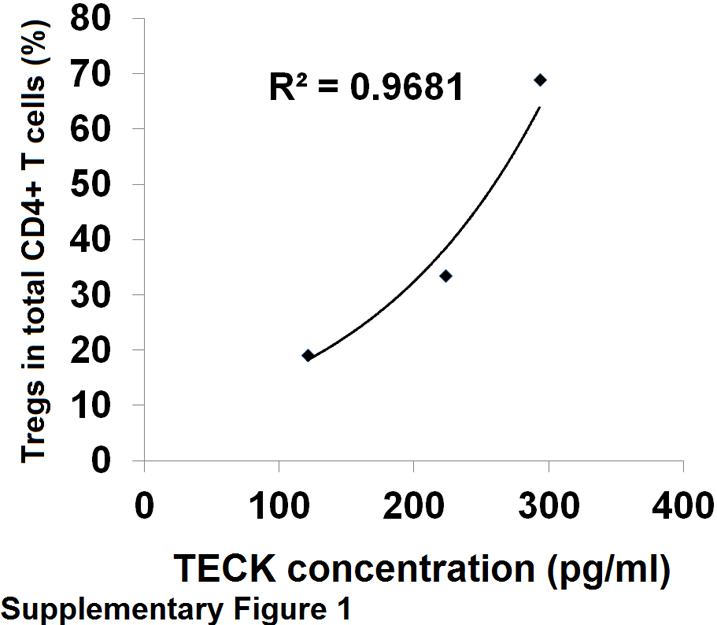

Supplement: Supplementary Figure 1 [file cddis2014414x2.tif]

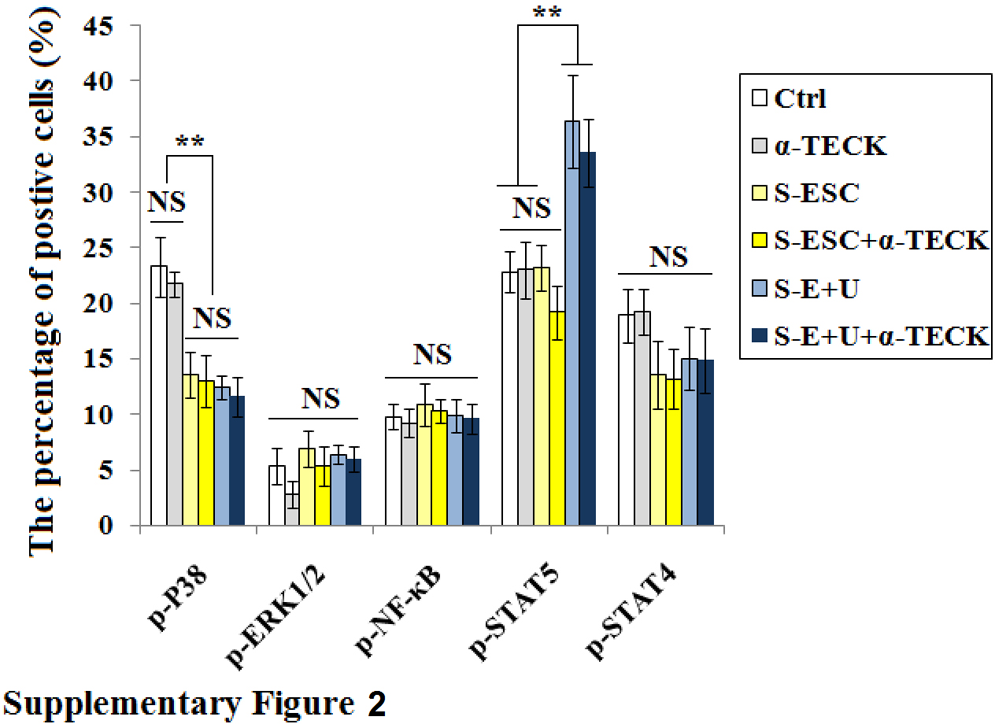

Supplement: Supplementary Figure 2 [file cddis2014414x3.tif]

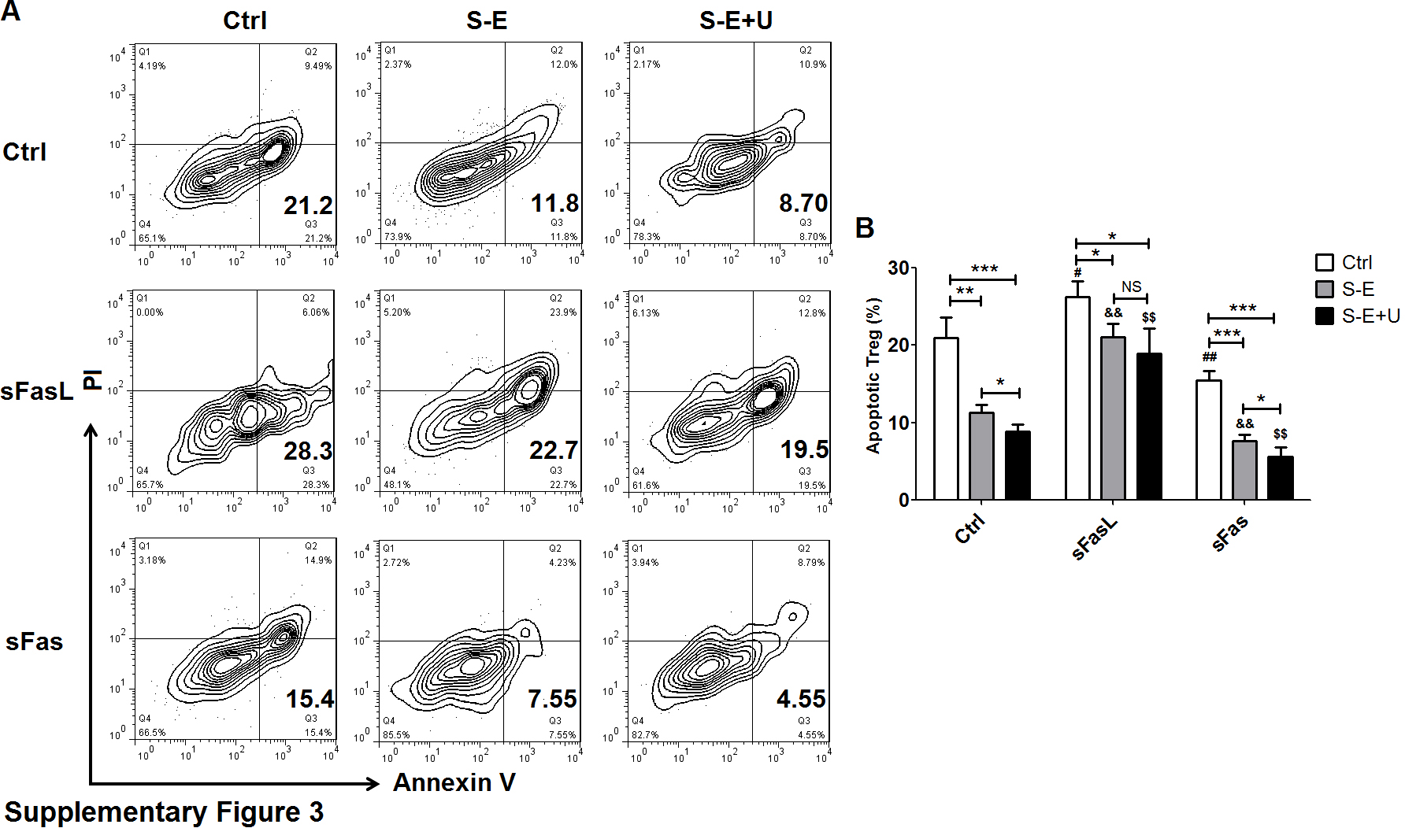

Supplement: Supplementary Figure 3 [file cddis2014414x4.tif]

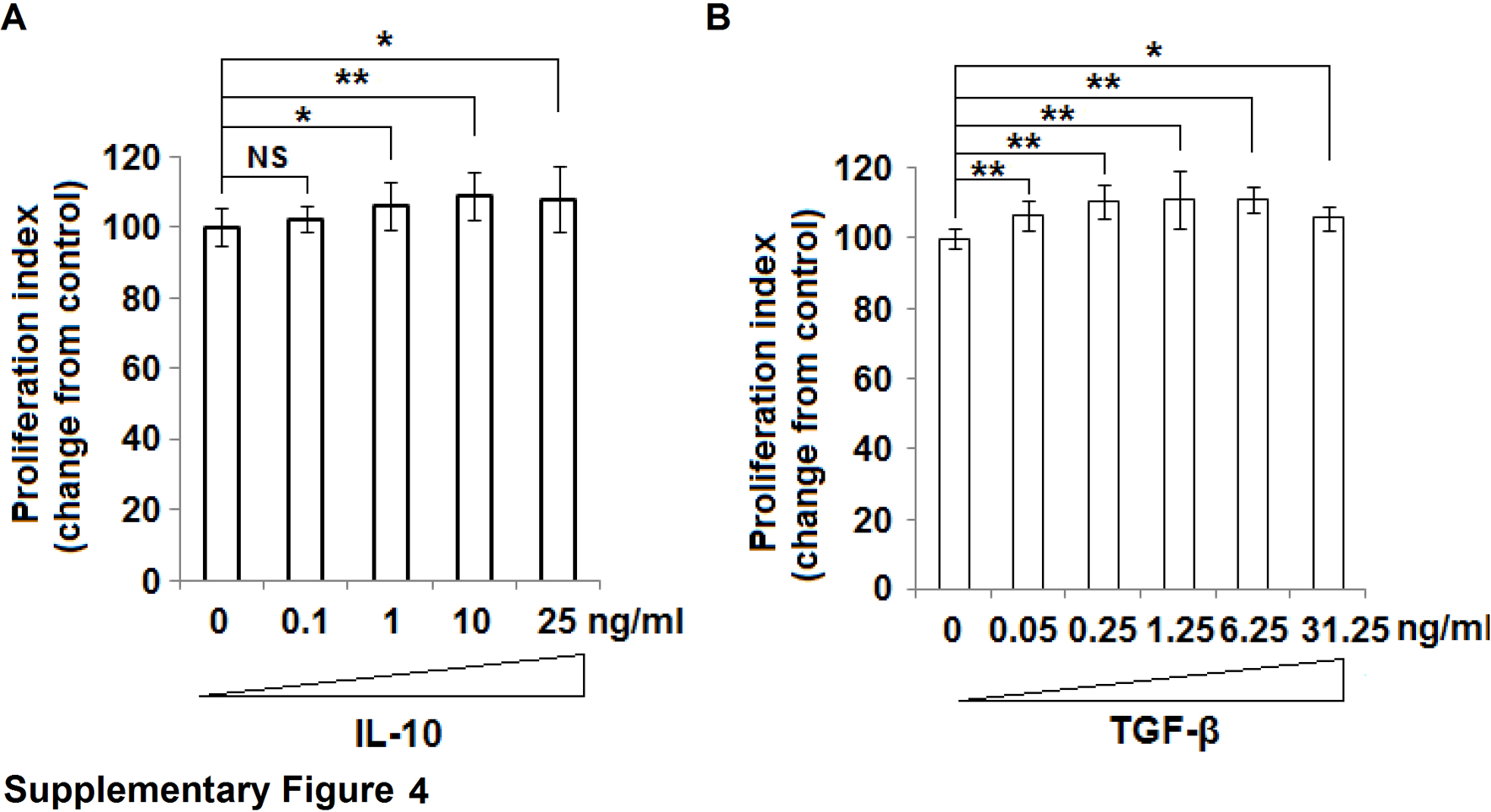

Supplement: Supplementary Figure 4 [file cddis2014414x5.tif]

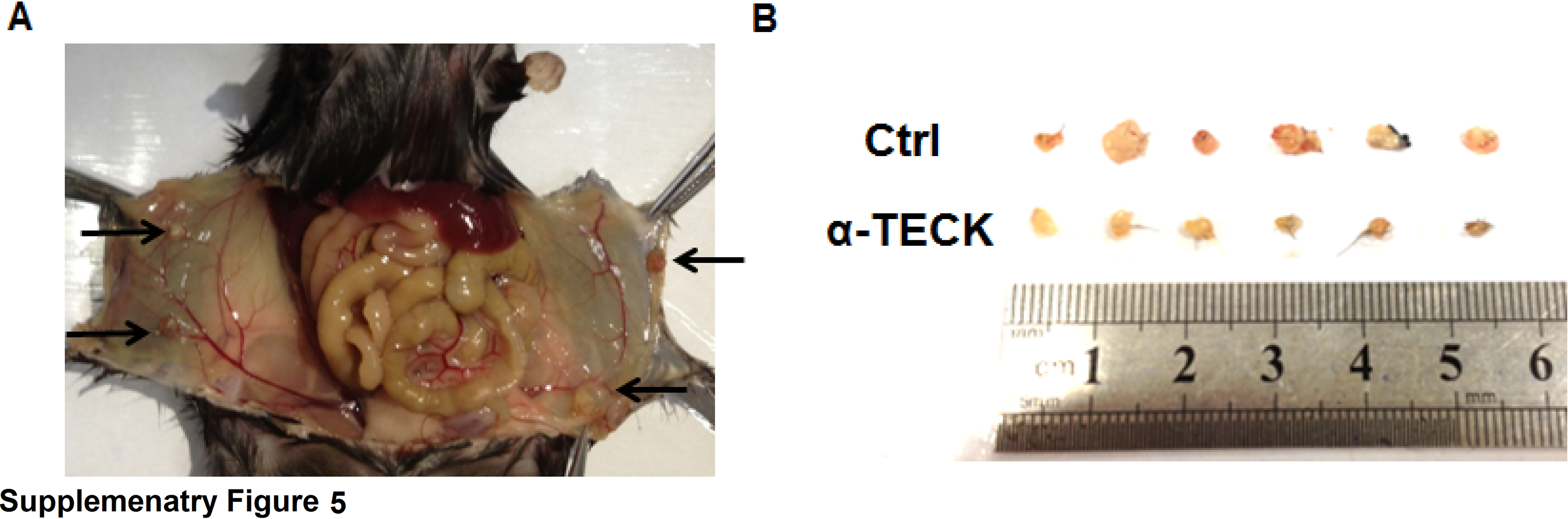

Supplement: Supplementary Figure 5 [file cddis2014414x6.tif]

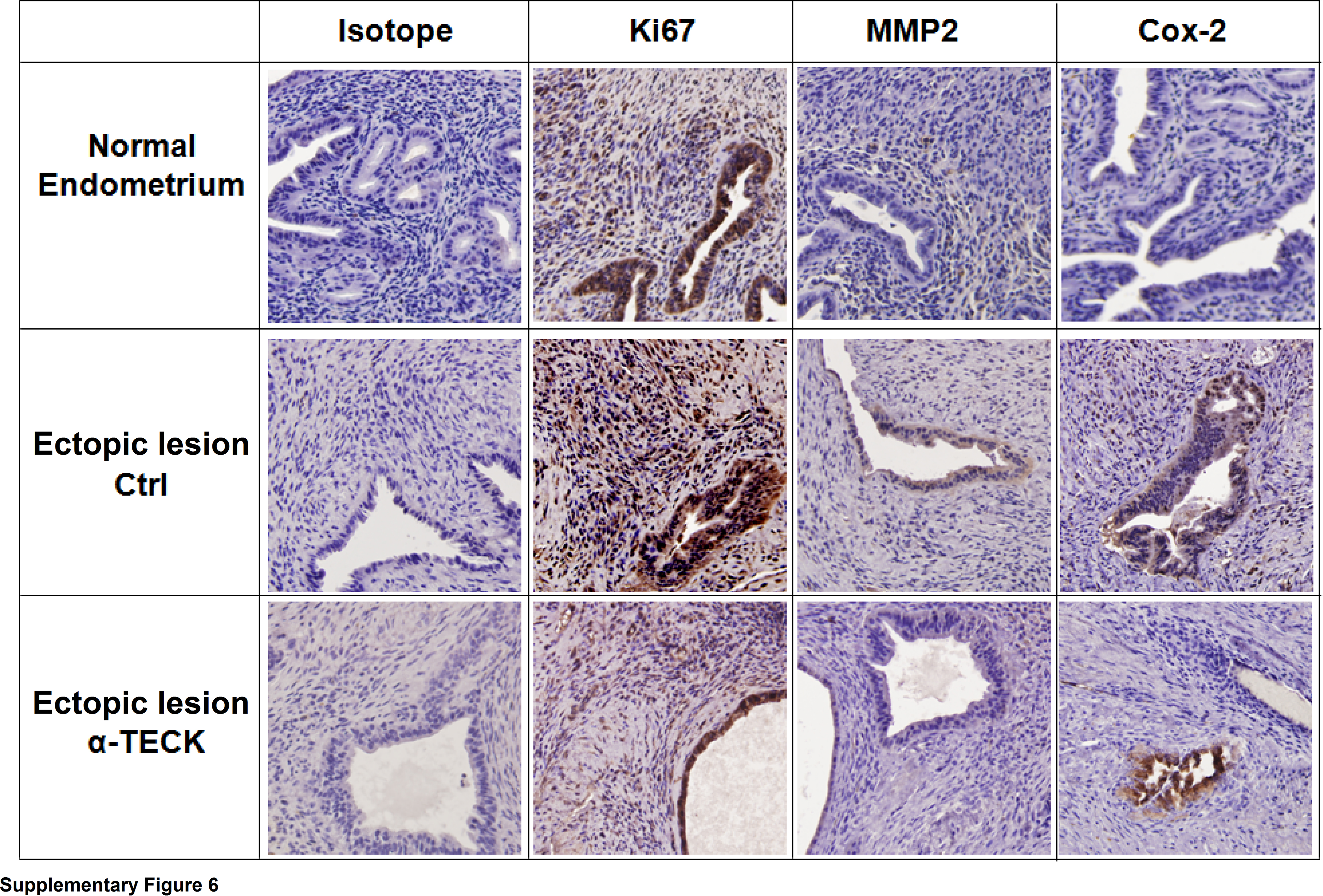

Supplement: Supplementary Figure 6 [file cddis2014414x7.tif]

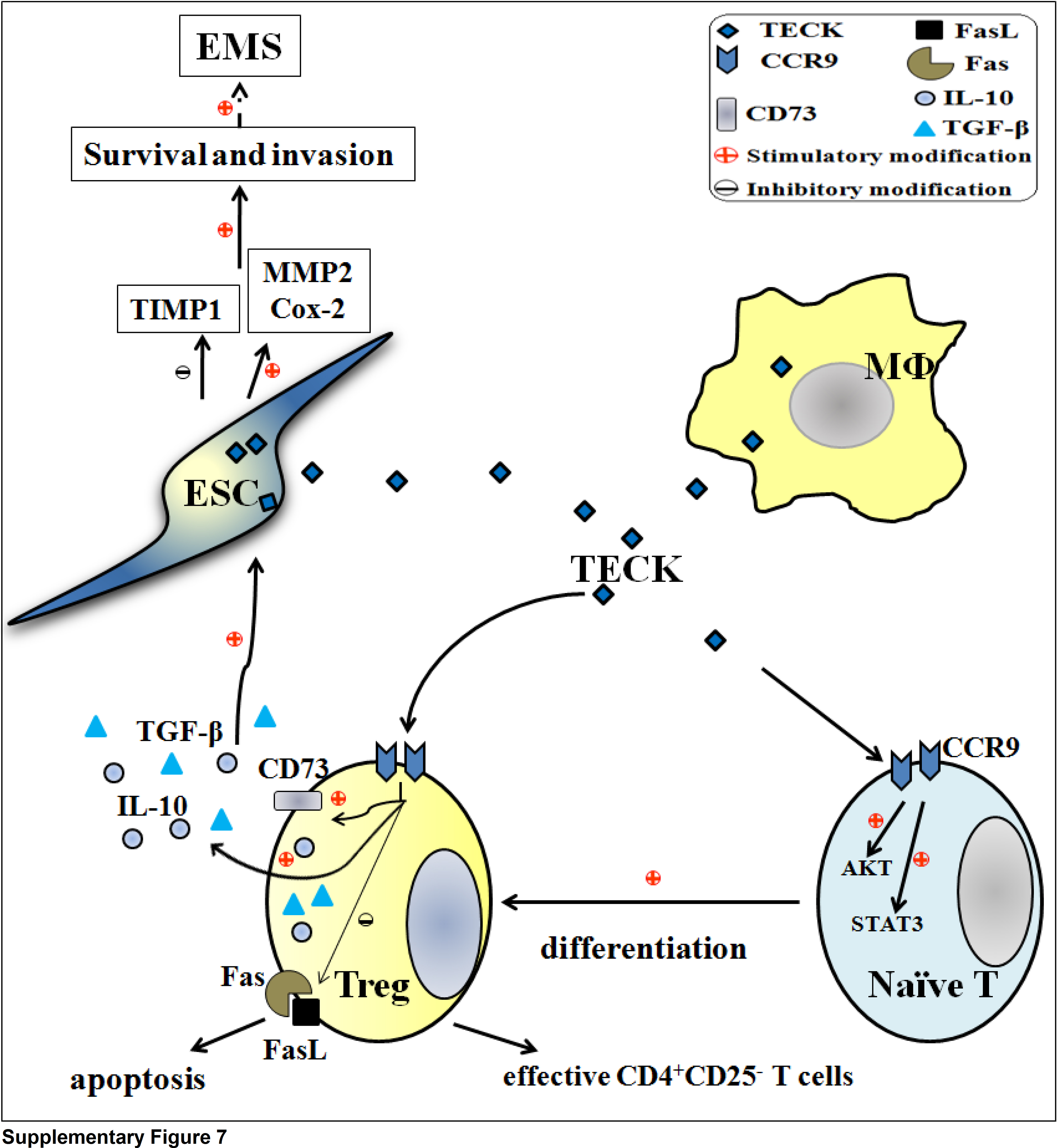

Supplement: Supplementary Figure 7 [file cddis2014414x8.tif]
